# Iterative alignment discovery of speech-associated neural activity

**DOI:** 10.1088/1741-2552/ad663c

**Published:** 2024-08-28

**Authors:** Qinwan Rabbani, Samyak Shah, Griffin Milsap, Matthew Fifer, Hynek Hermansky, Nathan Crone

**Affiliations:** 1 Department of Electrical and Computer Engineering, Johns Hopkins University, Baltimore, MD 21218, United States of America; 2 Department of Neurology, Johns Hopkins Medicine, Baltimore, MD 21287, United States of America; 3 Research and Exploratory Development Department, Johns Hopkins University Applied Physics Laboratory, Laurel, MD 20723, United States of America

**Keywords:** brain–computer interface, electrocorticography, speech, deep learning, voice activity detection, dynamic time warping, label discovery

## Abstract

*Objective*. Brain–computer interfaces (BCIs) have the potential to preserve or restore speech in patients with neurological disorders that weaken the muscles involved in speech production. However, successful training of low-latency speech synthesis and recognition models requires alignment of neural activity with intended phonetic or acoustic output with high temporal precision. This is particularly challenging in patients who cannot produce audible speech, as ground truth with which to pinpoint neural activity synchronized with speech is not available. *Approach*. In this study, we present a new iterative algorithm for neural voice activity detection (nVAD) called iterative alignment discovery dynamic time warping (IAD-DTW) that integrates DTW into the loss function of a deep neural network (DNN). The algorithm is designed to discover the alignment between a patient’s electrocorticographic (ECoG) neural responses and their attempts to speak during collection of data for training BCI decoders for speech synthesis and recognition. *Main results*. To demonstrate the effectiveness of the algorithm, we tested its accuracy in predicting the onset and duration of acoustic signals produced by able-bodied patients with intact speech undergoing short-term diagnostic ECoG recordings for epilepsy surgery. We simulated a lack of ground truth by randomly perturbing the temporal correspondence between neural activity and an initial single estimate for all speech onsets and durations. We examined the model’s ability to overcome these perturbations to estimate ground truth. IAD-DTW showed no notable degradation (<1% absolute decrease in accuracy) in performance in these simulations, even in the case of maximal misalignments between speech and silence. *Significance*. IAD-DTW is computationally inexpensive and can be easily integrated into existing DNN-based nVAD approaches, as it pertains only to the final loss computation. This approach makes it possible to train speech BCI algorithms using ECoG data from patients who are unable to produce audible speech, including those with Locked-In Syndrome.

## 1. Introduction

Restoring communication for people with Locked-in Syndrome (LIS) has recently become a viable goal for brain–computer interface (BCI) research [1, 2]. Previous work has demonstrated the feasibility of detecting and [3–6] and classifying [5, 7–11] speech from electrocorticographic (ECoG) [6, 11–13] and microelectrode array (MEA) [14] implants in patients with intact speech. In addition, audible speech may also be synthesized from ECoG [15–18] and MEAs [19]. Recent work has begun to translate these impressive results into recognizing [20–23] and synthesizing [23] speech in patients who are nearly or entirely unable to speak.

However, there remains a crucial need to detect the timing of speech attempts [24], even in the absence of microphonic ground truth, as with patients who are unable to produce audible speech. We refer to this task as neural voice activity detection (nVAD). Moreover, we distinguish between at least two different circumstances where nVAD may be used. First, online operation of a speech BCI may employ nVAD as an initial step before neural data is passed to a speech decoder for translation to text or synthetic speech. In this case, nVAD must detect speech attempts within unconstrained time periods, particularly in diverse and naturalistic contexts where operation of a speech BCI may occur outside the constraints of a controlled laboratory setting. Simple approaches such as template matching [5] and peak detection [25] may suffice here to detect speech attempts. Second, nVAD may be used for supervised training of downstream models for phoneme recognition and/or speech synthesis. Under these circumstances, precise synchronization between speech attempts and intended BCI output, at the level of phonemes, is essential. Spoken phonemes, the fundamental sound units of words, typically last around 100 milliseconds [25, 26], with consonant transitions as brief as 10 milliseconds [27]. Thus, even slight temporal misalignments will be detrimental to final model and user performance. Our approach targets this second use for nVAD in facilitating supervised model training when the precise timing of speech output is not available.

Normally, supervised model training for BCI relies on the segmentation of behavioral outputs, such as audible speech output or visible mouth movements, to provide the temporal boundaries of speech attempts [24]. Alternatively, task-level behavioral cues can attempt to constrain speech timing, but natural variations in response latency and production [28–31] limit their precision. On the other hand, alignment-free approaches, such as connectionist temporal classification (CTC) [32], allow model training without the need for strict 1:1 alignment between neural features and BCI output. However, their focus on optimization for completeness and correctness [32], without the additional objective of temporal precision, complicates their use for training models that provide immediate speech output.

The approach for nVAD that we take here determines the precise timing of neural activity associated with speech attempts in scenarios where the presence of neural voice activity within a broader time period is known, but the precise timing of transitions between speech and non-speech within the defined period are not. To support this, data must be collected in trials, with cues prompting the patient to attempt to speak the next word or phrase within a training set. We make a few simplifying assumptions about the problem statement: (1) The patient is cued to attempt speech within defined trial periods. (2) Behavioral statistics about the timing of the patient’s response to cues can be approximated. (3) An approximation of the durations of words within the set can be estimated. While relaxing these constraints to freely locate speech within unconstrained periods of silence is a highly desirable objective, we reserve that problem for future efforts.

We call our approach ‘iterative alignment discovery dynamic time-warping’ (IAD-DTW), as it utilizes a fully differentiable DTW algorithm to learn speech onsets, then speech durations, before it eventually converges on labels for individual trials. IAD-DTW is an iterative procedure that relies on the backpropagation algorithm to identify neural patterns corresponding to speech activity, with a focus on the 2-class problem of speech vs non-speech. To achieve this, the procedure currently requires, in addition to the previous assumptions: (1) a cued task design with one attempted utterance (e.g. single words or syllables) per trial and (2) classifiable patterns of neural activity associated with speech vs non-speech segments. To test the performance of IAD-DTW, we applied the algorithm to ECoG data from epilepsy surgery patients with intact speech. In these experiments, we simulated the absence of ground-truth speech labels. After the model was initialized with an estimate of speech onsets and durations across trials, we systematically shifted these priors relative to neural activity in ECoG recordings to test IAD-DTW’s capability to localize and refine nVAD boundaries at varying levels of difficulty.

## 2. Method

### 2.1. Neural data

We analyzed ECoG data originally used in two previously published studies, including one from a published dataset distributed across two papers from University of California, San Francisco [11, 33] and one from John Hopkins University [5]; both datasets are available online. In total, we analyzed data from three patients (P1, P2, P3) implanted with subdural ECoG grids over dominant language hemisphere to guide surgical treatment of drug-resistant epilepsy. Each patient was implanted with one high-density electrode grid covering ventral sensorimotor cortex (vSMC) and superior temporal gyrus (STG) with 4 mm electrode spacing, with row and column dimensions of 16 × 16 for P1 and P2 [11, 33], and 16 × 8 for P3 [5]. Further clinical details, including electrode maps superimposed onto the participants’ anatomies, are available in the corresponding publications. These datasets were selected based on electrode coverage of anatomical speech areas to ensure ideal nVAD performance, to sufficiently benchmark our proposed method. P3 was included to test model performance with smaller amounts of data and less comprehensive anatomical coverage.

### 2.2. Experiment task design

All patients consented to voluntarily participate in a prompted speech task in which each patient spoke a variety of consonant-vowel (CV) syllables. P1 and P2 spoke 57 CVs (19 consonants × 3 vowels), between 15 and 100 times, each in response to a textual prompt, approximately every 2 s with unspecified inter-trial intervals [11, 33]. P3 spoke 12 CVs (4 consonants × 3 vowels), 10 times each, in response to a textual prompt, and 10 times each, in response to an acoustic prompt, approximately every 4–5 s with a uniform inter-trial jitter between 1 and 2 s [5]. Patients were instructed to speak immediately upon reading or hearing the received cue [5, 11, 33]. All ground-truth speech signal onsets and offsets were labeled by human annotators at both centers [5, 11, 33].

### 2.3. Signal processing

Neural data was resampled from 3052 Hz for P1 and P2 to 1000 Hz to match P3. Data was re-referenced to remove common mode noise via bipolar derivations along the horizontal and vertical dimensions of the grid [13]. We additionally padded the edges of the arrays to avoid resizing the grids with bipolar re-referencing and pooled signals from both the horizontal and vertical re-reference. Edge cases were resolved by padding to maintain the spatial dimensions of the ECoG grids.

Next, we obtained the signal power envelopes of the neural signals by applying a bank of second-order Infinite Impulse Response (IIR) filters to the signals, with a frequency range of 0–250 Hz, 20 Hz spacing, and 20 Hz bandwidth, which we chose to minimize the resulting delay induced on the filtered signal output. This yielded the following frequency ranges for each filter, where the DC filter was a half-width low-pass filter with filter quality matching the bandpass filters: 0–10, 10–30, 30–50, 50–70, 70–90, 90–110, 110–130, 130–150, 150–170, 170–190, 190–210, 210–230, and 230–250 Hz. The resulting envelopes were squared and then smoothed using a second-order IIR low-pass filter with a cutoff frequency of 10 Hz. We then downsample the power envelopes to 100 Hz, resulting in 10 millisecond frames [6]. Incorporating data from all frequency bands avoids excessively relying on any single frequency range. While high gamma frequencies have traditionally played a significant role in BCI performance for overt speech tasks (as utilized in the simulations in this study), recent research suggests that lower frequencies are crucial for silent speech [21, 34], which constitutes the primary focus of our approach. Squaring the envelope served to increase the detectability of relevant neural events relative to noise [35].

We chose narrow bandwidths for the filters so that they would converge toward the squared amplitude of the analytic signal used in previous studies [11], while still being computationally efficient for real-time speech decoding. Additionally, we minimized the filter order to minimize delay. To ensure real-time compatibility, we applied the filters causally, moving only forward in time. We did, however, apply a consistent delay (of 25 ms) to each band to approximately account for frequency-dependent phase distortions incurred by the IIR filters, as further described within the supplementary materials.

The squared envelope trajectories [35] were then normalized to zero mean and unit variance with respect to silent periods pooled across the beginnings and ends of recordings [11], which always contained at least 10 s of silence. Using silent periods at the beginning of each session allows our approach to be compatible with real-time speech decoding models [36].

To test the efficacy of our approach, we used hand-transcribed annotations of the patients’ synchronized microphone recordings to generate corresponding binary (speech/non-speech) labels for each frame of neural data. These labels were not used by our proposed approach during nVAD model training but only provided a ground-truth reference for subsequently evaluating model performance.

### 2.4. Modeling approach

We repurposed a DenseNet architecture, originally designed for RGB image classification [37], to classify (or ‘decode’) neural voice activity by utilizing spatial information from neighboring electrodes arranged in a 2D grid. Here, the *X* and *Y* axes of the image given to the network are the rows and columns of the 2D grid, and the 3 RGB color channels are instead replaced by 13 frequency bands. We do not incorporate time into the network’s convolutions. Instead, we input individual 10 ms frames or ‘static images’ into the network, in order to reduce the latency of the nVAD detector. Similar to a previous implementation [16], our DenseNet model included an initial convolutional layer, followed by three dense blocks with transition layers (except for the final block) and finally a single global average pooling and linear prediction layer. The size and number of network layers matched the previous implementation [15], except we predicted 2 (class) outputs instead of 40 (mel) outputs, and we opted for spatial dropout [38] rather than ordinary dropout.

**Figure 1.**
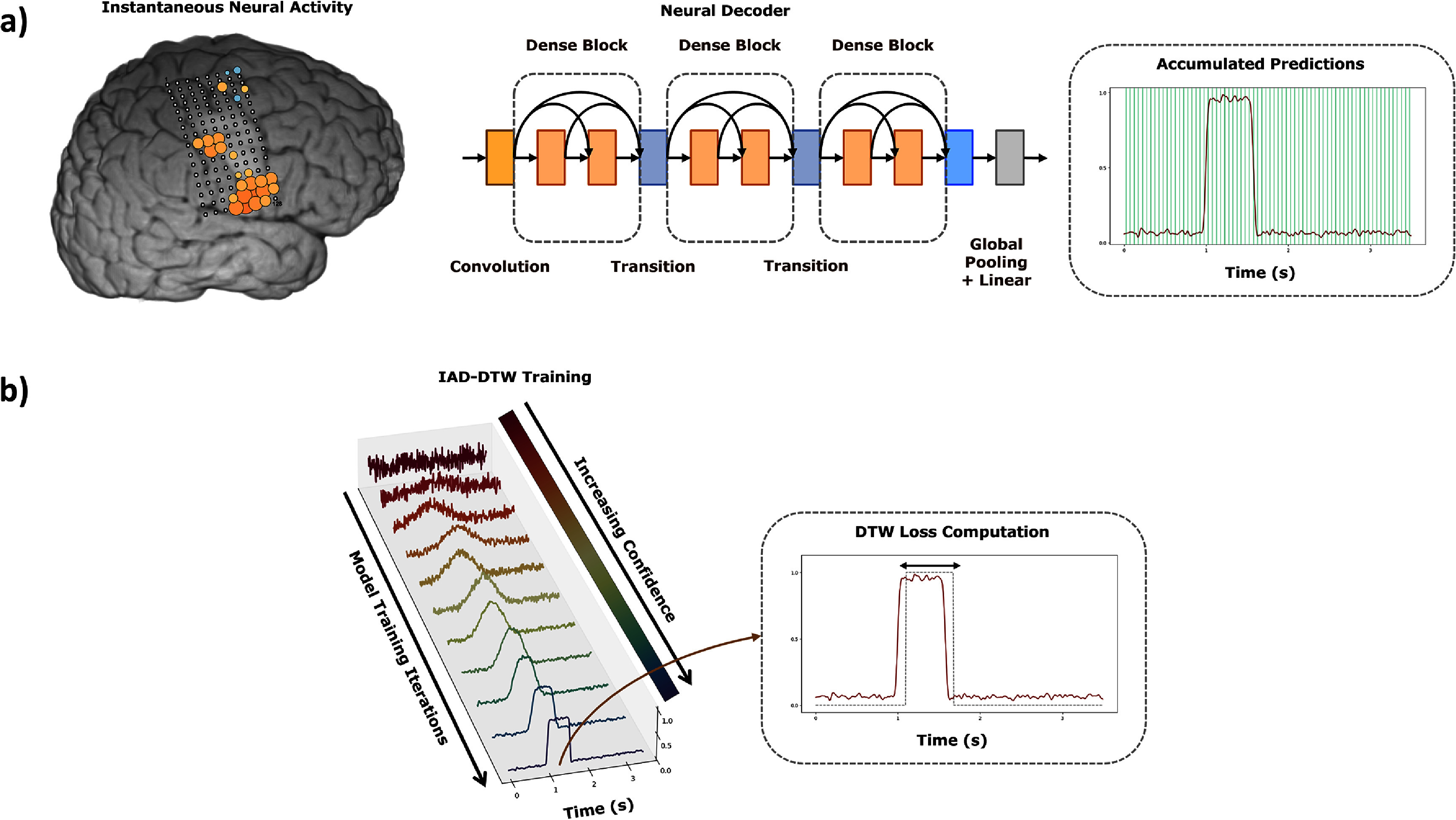
Overview of the Iterative Alignment Discovery Dynamic Time Warping (IAD-DTW) approach for neural voice activity detection (nVAD). (a) Instantaneous neural activity from P3’s brain (schematized from average activations in top left brain image) is processed frame by frame every 10 ms by a DenseNet neural decoding model (top middle), which evaluates spatial patterns within the received neural data to predict speech likelihood (top right plot, speech likelihood plotted in red) sampled (top right plot, solid green lines) for each trial of speech training data. (b) As IAD-DTW training proceeds, a series of predictions of speech likelihood are refined for each trial. This process is illustrated (bottom left plot) for a single trial of neural data as model training progresses. In each training iteration, IAD-DTW carries over the outcome of previous iterations for the onset and duration of speech-associated neural activity for a given trial, which is stored internally as a target binary label trace (bottom right plot, thin gray dotted line) and aligned with the model predictions (bottom right plot, solid red line) via DTW during model training.

Figure 1 provides an overview of an overview of the nVAD approach used in this study. In this approach, neural signals are first converted into a sequence of spatially arranged features and processed by a DenseNet neural decoding model to predict time windows within the trial that indicate speech activity. During model training, the outcome of previous predictions (a label trace with what the model believes to be the best current labeling of each trial) and current predictions for each trial are realigned using dynamic time warping (DTW), and the difference between the previous and current predictions is calculated as the loss. The backpropagation algorithm is then used to update the model’s weights based on this loss. In parallel, we update our label trace to match the optimal onset and duration (with the lowest loss) suggested by the current iteration for use in the next iteration. As training proceeds, the model becomes increasingly adept at identifying common spatial patterns of neural activity associated with speech. In tandem, it updates its labeling of each trial of neural data as it learns to better segment the time windows associated with speech, which is likewise reflected in the confidence of the model outputs.

P1, P2 and P3 had 2628, 1645 and 240 trials worth of data, respectively. For our training hyperparameters, we used a batch size of 4 trials (with one cued utterance per trial) and an Adam optimizer with a learning rate of 5 × 10^−4^. We applied sample weights during model fitting to correct for class imbalance. To prevent overfitting, we applied an l2 penalty of 1 × 10^−4^ to each layer’s weights and a spatial dropout of 0.1 for regularization. Additionally, we employed a learning rate scheduler that reduced the learning rate upon validation loss plateauing. We performed a 5-fold cross-validation, where each model was trained for 80 epochs. This resulted in an 80-20 cross-validation split, or 2102, 1316 and 192 trials for training for P1, P2 and P3, respectively.

While ground-truth labels were used to validate the convergence of the DenseNet neural decoding model during subsequent simulations, they were strictly separated from any tuning of the network, such as the learning rate at each training step. Instead, the loss for the expected labels was used as the only proxy available to evaluate the network’s ability to generalize.

### 2.5. Simulation assumptions

Our approach aims to predict the time window during which patients speak or attempt to speak within single trials of a cued speech task, using only neural activity (designated by 1 or 0 for speech or non-speech). To simulate the challenges posed when ground truth is unavailable, as in the case of a patient unable to produce overt speech, we withheld ground-truth labels from model training. We assumed, however, that certain statistical priors, such as the average latencies and durations of reference speech from subjects with intact speech could allow a rough guess as to when speech might have been attempted within each trial. Since we lacked access to a parametric model of the neural activity itself, we approximated this in our simulation by modifying the trial-wise data. Typically, patients exhibit variable response latencies and durations during speech attempts, and these statistics are reflected in the underlying neural activity. To account for this, we first realigned the data to response onset before adjusting it to match each simulation condition. Subsequently, we jittered, padded, and linearly stretched the data to align with the expected statistics of the simulation, rather than those from the original data collection. This task and subject-independent simulation procedure eliminated the impact of auditory or visual cues and variable or fixed inter-trial intervals through the response alignment step. With these steps, we enforced response timings with a consistent, unimodal distribution—the typical distribution of response timings—regardless of the source dataset used in each simulation.

Through our simulations, we quantified the degradation of performance due to trial-wise misalignments between ground-truth labels and labels derived purely from neural activity. We also compared our approach to blind labeling approaches with fixed latency and duration (which we later consider ‘naïve model assumptions’) to infer what might happen when speech timings are unreliable or entirely absent, as in patients who cannot produce audible speech. Furthermore, we used high-quality, hand-annotated, audio-derived labels to determine the highest possible, or ‘oracle’, performance that would be expected if the model had access to them during traditional supervised training [6].

Collectively, loosely initialized nVAD labels across all trials can provide a parameterized distribution that can be used to initialize a search for labels within trials. Instead of modeling all time points within a trial independently, we focused on the two most salient variables: the onset of neural voice activity (relative to trial start) and the duration of neural voice activity. We modeled these variables as normally distributed, meaning they followed a Gaussian distribution with some mean and standard deviation, to describe the likelihood of patient voice activity spanning a certain period of each trial.

**Figure 2.**
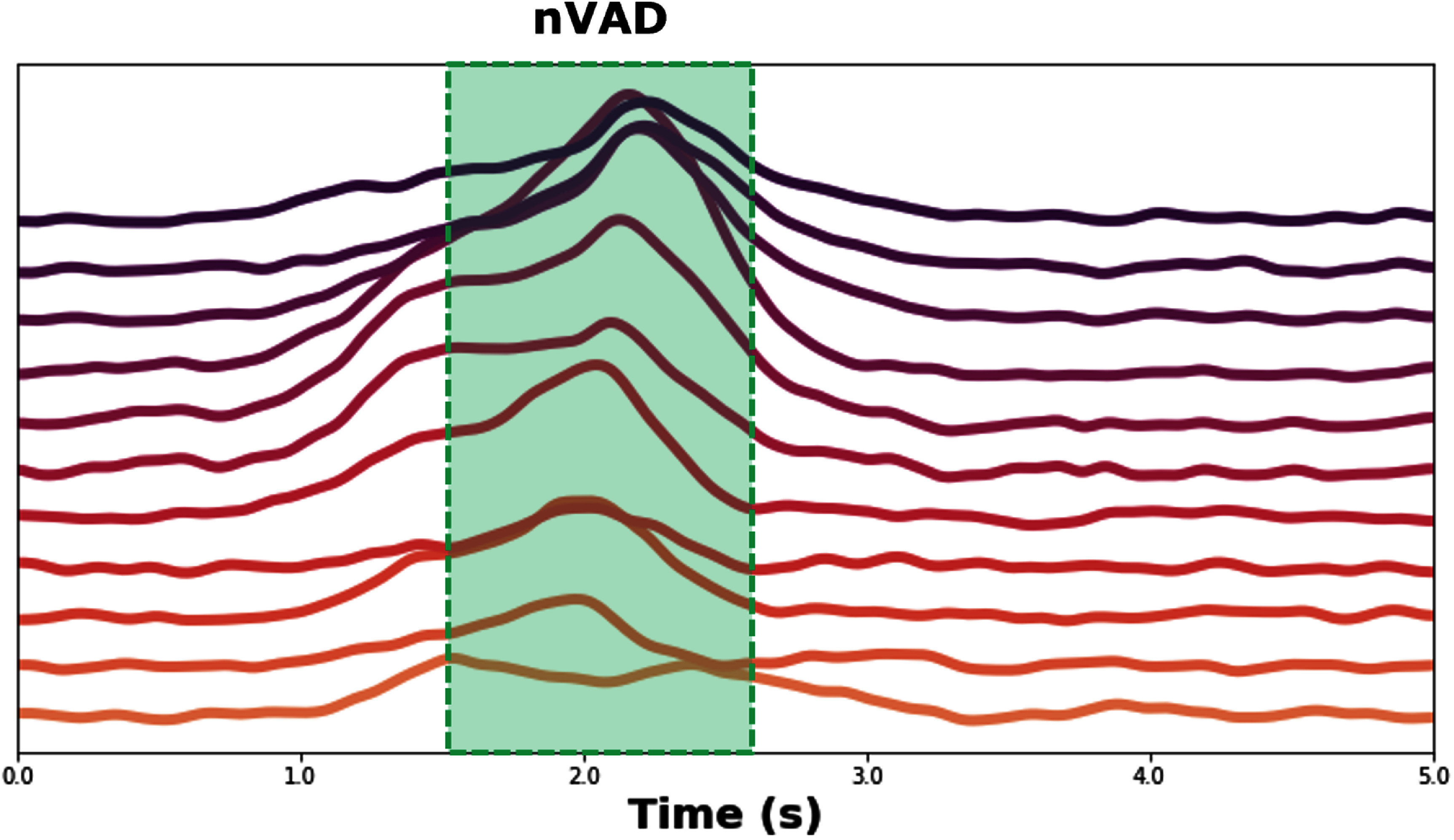
Timing variations in neural responses before and during speech. Neural responses to speech from P3 are shown to illustrate how the timing of neural responses relative to speech can vary substantially across different ECoG recording sites, posing a challenge for methods, like ours, attempting to infer the onset and duration of speech from neural responses alone. Log-transformed high gamma power was averaged across all 240 trials and smoothed for individual ECoG recording channels (rows) selected for this illustration. Variations in the timing of neural activity among individual recording sites may reflect different sensitivities to preparation and execution of the articulatory gestures necessary for speech. This underscores the necessity for algorithms like IAD-DTW that can learn from many trials the spatial-temporal patterns of neural activation that most consistently correspond to putative speech and iteratively apply these to find the best fit for individual trials of a cued speech task. Learning these patterns and applying them would be difficult, if not impossible, for human annotation.

Studies have shown that speech-associated neural activity typically follows the response latencies of the speech itself [35]. In the case of patients with paralysis who cannot produce audible speech, we hypothesize that their attempts to speak will still elicit neural activity patterns that are distinct from other neural signals (as elaborated in figure 2 above). While these attempts may be challenging to detect, we assume that the distribution of response latencies for these attempts will resemble that of the patient’s overt speech before paralysis (if available), that of a reference speaker (if appropriate), or can be obtained from average high-gamma activations with sufficient signal-to-noise ratio.

### 2.6. IAD-DTW algorithm

In traditional supervised training of a classifier, a direct mapping of inputs to class labels is crucial for accurate classification. These labels are typically of high quality and accuracy, allowing the classification model to converge based on distinctions between classes. While this model may not achieve perfect prediction and categorization, its convergence relies on the separability of inputs from each class. However, in scenarios where label reliability is compromised, as when spoken acoustics are not available and can only be approximated based on the constraints of a trial-based task design, training a model with imperfect labels can lead to a ‘naïve’ classifier. The term ‘naïve’ refers to the classifier’s inherent inability to find correct labels. Instead, it strives to fit imperfect labels to the best of its capability. We use this ‘naïve classifier’ as our baseline, which we compare to more advanced approaches, because it is the simplest and most straightforward approach to the problem.

Alternatively, IAD-DTW is a data-driven approach that optimizes nVAD predictions at the frame level, without requiring prior knowledge of label alignment with the neural activity. To accomplish this, it stochastically searches for shifts between the input and output during the initial model training and later optimizes the duration of the target. The design of IAD-DTW is largely motivated by the assumption that onset and duration are the most important variables for describing variations in the detected speech output.

To optimize the predicted labels, we first needed to select an appropriate binary classification (or ‘detection’) loss. In this study, we chose a modified version of KL-divergence called ‘label relaxation’ [39], which allows for small deviations from the target without incurring penalties. We set the *α* hyperparameter to 0.15 and used it as the frame-wise metric for all subsequent methods. When using a naïve one-to-one classification loss, label relaxation loss is computed directly on the prediction and target without realignment. DTW improves upon this by querying each possible prediction-target pairing to find a new, optimal alignment path with the lowest loss.

Rather than optimizing based solely on the single best alignment path, we adapted the Soft-DTW algorithm [40] with its non-negative divergence extension [41] to ensure that the global minimum loss occurred when the prediction exactly matched the target. We used the default settings for this algorithm but made a slight modification to allow the use of relaxed KL-divergence as a valid divergence within this newer variant of Soft-DTW, which is described in detail in the supplementary materials.

**Figure 3.**
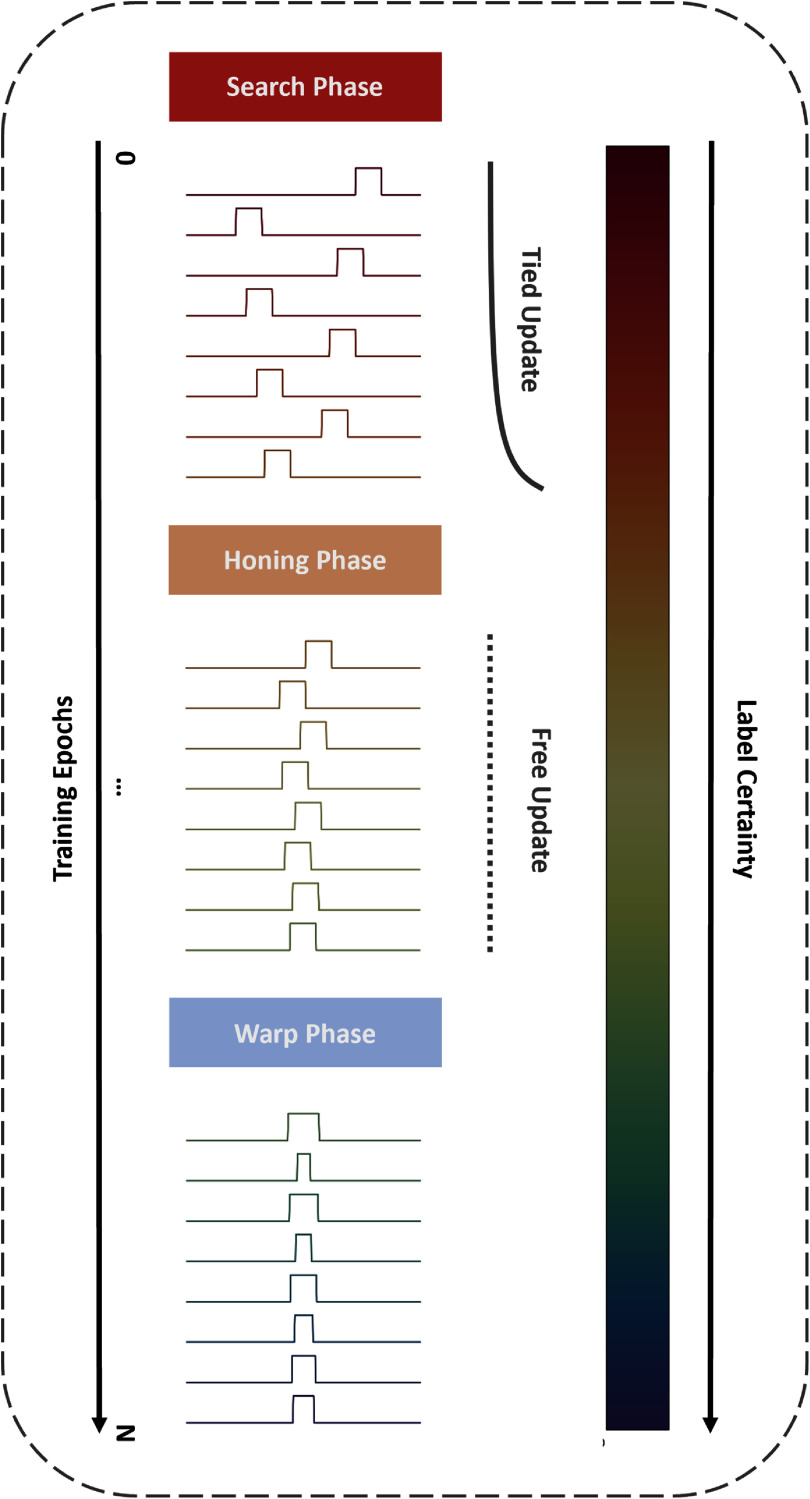
The Iterative Alignment Discovery Dynamic Time Warping (IAD-DTW) algorithm. IAD-DTW is an iterative process that localizes neural activity corresponding to speech, as shown by the binary neural voice activity detection (nVAD) label traces above. Here, we depict the convergence for a single idealized trial within each phase of the IAD-DTW method, across training epochs (moving downward), as the network gains more confidence and gradually settles on the final label set. Initially, IAD-DTW localizes the speech-associated neural activity with fixed durations before ultimately fine-tuning it and determining the optimal boundaries. As the initial nVAD predictions can be noisy, gradient updates are exponentially smoothed across training epochs for a given trial, effectively tying these normally independent updates together, before allowing the network to update freely in the next phase. After this point, the network has settled on approximate locations for the nVAD label traces, and the boundaries can be optimized. At the end of training, the optimal label set can be retrieved by taking the maximum-likelihood DTW warp paths.

With these building blocks and previous assumptions at our disposal, we decided to separate model training into three phases of varying complexity (illustrated in figure 3 above), as the network initially discovered the onset and then the duration of the nVAD alignment.

(1) Firstly, in the ‘search’ phase, the model searches for the rough locations of neural activity by using a custom sliding DTW algorithm, referred to as ‘Slide-DTW’. Slide-DTW directly compares a reference sequence (here the best current label pattern) by sliding it along another sequence (here the model prediction) without warping the reference. In this way, the network efficiently tests each shift or ‘response latency’ without needing to also test for the duration (since the reference pattern is fixed).
  (i) To avoid catastrophic misfitting, we also used an exponential moving average (EMA) tying mechanism that smooths gradient updates across training epochs by using the outcome of predictions from each trial in the previous training iterations as a statistical prior for the alignment optimization in subsequent training iterations independently for each trial.
(2) Secondly, in the ‘honing’ phase, the initially discovered locations of neural activity are refined using Slide-DTW, which is initialized with the same labels as the previous phase. In this phase, Slide-DTW is run without the EMA tying mechanism, allowing the model to freely and rapidly adjust its alignment hypothesis for the response latencies without constraint.
(3) Thirdly, in the ‘warping’ phase, the durations of neural activity are optimized. We initialize the labels with optimal response latencies from the previous phase, if available. For our standard DTW training, this is the only phase, regardless of whether the labels are misaligned or not. This phase allows the model to learn the optimal response durations based on prior probabilities obtained from the previous labels. At the end, we obtain the maximum-likelihood labels from Soft-DTW.

The IAD-DTW algorithm incorporates two, distinct and independent, approximate priors, one for duration and another for shift, which we estimate based on the outcome of previous model predictions. The duration prior relies on the natural bias of DTW towards ‘diagonal alignment,’ signifying a one-to-one alignment between each sequence (the model prediction and training target). Deviation from this default alignment occurs when a compelling reason exists, typically driven by a strong incentive that reduces alignment cost, despite extending the alignment path. DTW incurs this implicit penalty since the cost along the alignment path is summed, not averaged. By positioning the labels at the best current guess of the speech’s position within a trial, a loose duration prior is created, which is depicted in figure 1. On the other hand, the shift prior is more explicit, representing the likelihood of each possible shift when sliding the labels of a fixed length along the model prediction. These likelihoods are approximated from the loss obtained for each shift in a procedure that we outline in further detail within the supplementary materials. The behavior of these likelihoods over the course of model training is depicted in figure 6.

Combining these principles, in the initial stages of IAD-DTW, the labeling pattern is allowed to shift forward and backward in the search for the optimal position, updated internally via the shift prior mechanism. Initially, these priors are smoothed via an EMA for a set number of epochs, and then they are allowed to update freely for another set number of epochs. Once the optimal position is determined, it is fixed, and the model is only allowed to ‘warp’ the labeling pattern, involving stretching and contracting with the assistance of DTW, following our duration prior mechanism for the remaining epochs.

Overall, IAD-DTW is a modified version of standard DTW training, which optimizes nVAD frame-wise predictions without initially knowing the alignment of the labels with the neural activity. By stochastically searching shifts between the input and output early during model training before optimizing the duration of the target later during model training, IAD-DTW allows the model to gradually latch onto the final label set, effectively discovering the onset and duration of the nVAD alignment in an iterative manner. However, as with all DTW training, IAD-DTW only modifies the backpropagation step of model training. Any real-time-capable model, once trained, can still be applied causally to an unseen sequence of neural data (e.g. sample by sample or within a sliding window).

The durations of these IAD-DTW phases were selected empirically. Here, we chose 10, 20 and 50 training epochs for each phase, respectively, for all experiments in which IAD-DTW was applied. In general, the initial phases completed relatively quickly, so most of the training time was allocated toward the final stage, in which the learned labels could simply be further fine-tuned.

### 2.7. Model evaluation

We performed two simulations, Simulation A and Simulation B, to investigate the effects of local and global misalignments when no speech labels are available. In both simulations, the ground-truth timings were randomly jittered by drawing samples from a normal distribution, to artificially reconstruct trials with the desired response latencies and durations. To achieve this, we padded and interpolated the neural data samples to recreate the desired shifts and durations in a procedure that is detailed further in the supplementary materials. However, in Simulation A, we evaluated the impact of label jitter without shift, which involved increasing the variance while keeping the overall mean onset of the labels unchanged. On the other hand, in Simulation B, we assessed the impact of label shift with moderate jitter, which involved shifting the mean onset in increasing amounts while maintaining a constant variance.

To simplify the number of simulation conditions, we increased the standard deviation of both speech onset and duration jointly in Simulation A by doubling their values for nine different conditions. For Simulation B, we kept the standard deviation of speech onset and duration at a moderate level and varied the mean speech onset in doublings, resulting in an additional nine conditions. For all conditions, we fixed the target label durations to the median duration of voice activity for each subject (P1: 460 ms, P2: 370 ms, P3: 600 ms) to remove the factor of label misalignment as much as possible from our evaluation of ideal performance. To accommodate the varying tasks rates, we segmented data on a per patient basis into trials of uniform lengths (P1: 1.5 s, P2: 2.0 s, P3: 3.5 s) and uniform speech onsets (P1: 0.5 s, P2: 1.0 s, P3: 1.0 s). We compared the nVAD accuracy achieved by three methods: (1) a naïve model trained with a direct one-to-one binary classification loss directly on the mismatched expectation, (2) a standard DTW loss on a [prediction × target] matrix of all possible losses, and (3) our proposed IAD-DTW loss extension.

To generate more accurate error bounds, we applied 5-fold cross-validation and ran each fold 5 times each, resulting in 25 values for each simulation condition (each point in figure 6). We computed independent oracle and chance accuracies for each patient to account for variability in data decoding quality and speech rate. Oracle performance refers to the performance of a standard model with access to the ground-truth labels for voice activity prediction, which therefore is dependent only on the quality of the data itself and the model’s ability to capture it. Chance performance, on the other hand, represents the best possible performance of a ‘null classifier,’ achieved by labeling everything as the majority class, which in this case is silence. Finally, we highlighted detected neural voice activity periods obtained through a simple, recursive, two-threshold filtering algorithm with minimum 150 ms speech duration and 100 ms rest thresholds to illustrate the detectability of obtained prediction probabilities across epochs. Note that all accuracies were nonetheless computed without the aid of this approach. Further details on this algorithm may be found in the supplementary materials.

## 3. Results

### 3.1. Accuracy under simulated misalignment of speech and speech-associated neural activity

**Figure 4.**
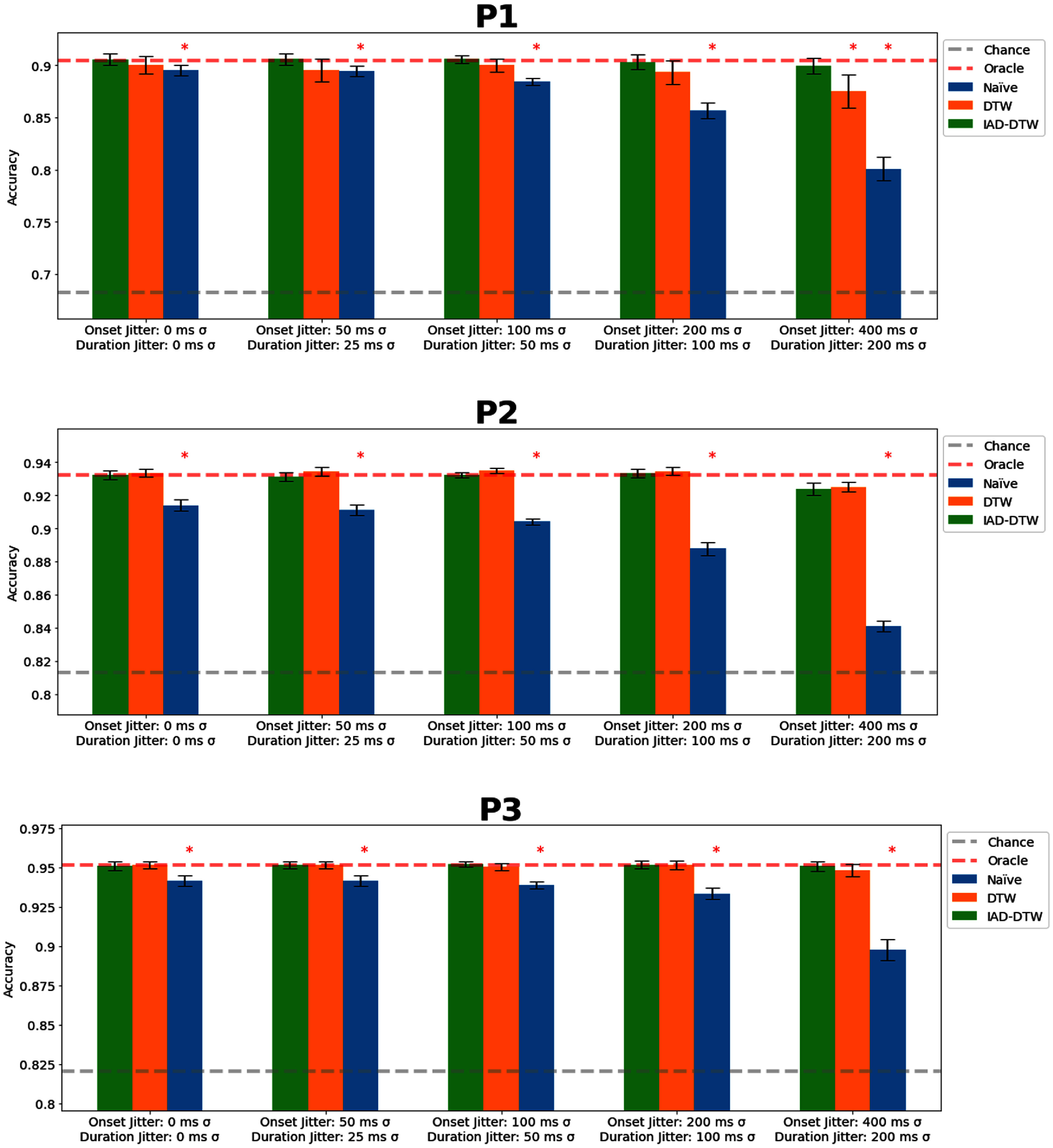
Simulation A—Zero-mean label distortion. Here, we measure the robustness of naïve (standard, direct classification), dynamic time warping (DTW) and iterative alignment DTW (IAD-DTW) to increasing amounts of variance in the unknown response distribution without shifts. Statistically significant differences in performance relative to IAD-DTW (ANOVA, *p*-value <= 0.01) are marked by asterisks.

In our first simulation (figure 4), we simulated data jittered around a known response latency. Here, we tested the relative robustness of all DTW-based procedures, even to extreme jitter exceeding typical onset latencies. Performance was relatively flat (<1% absolute change in accuracy) even at large jitters. In contrast, a naïve classifier without the capability for realignment performed increasingly poorly with increasing amounts of jitter, with performance particularly impacted past 100 ms onset jitter and 50 ms duration jitter (fell by >3% to as much as ∼10%, though remaining above chance). Even at moderate 100 ms onset jitter, close to what may be observed in practice, DTW provided a notable performance boost by preserving the ideal accuracy. This demonstrates the major utility of realignment for noisy labels.

**Figure 5.**
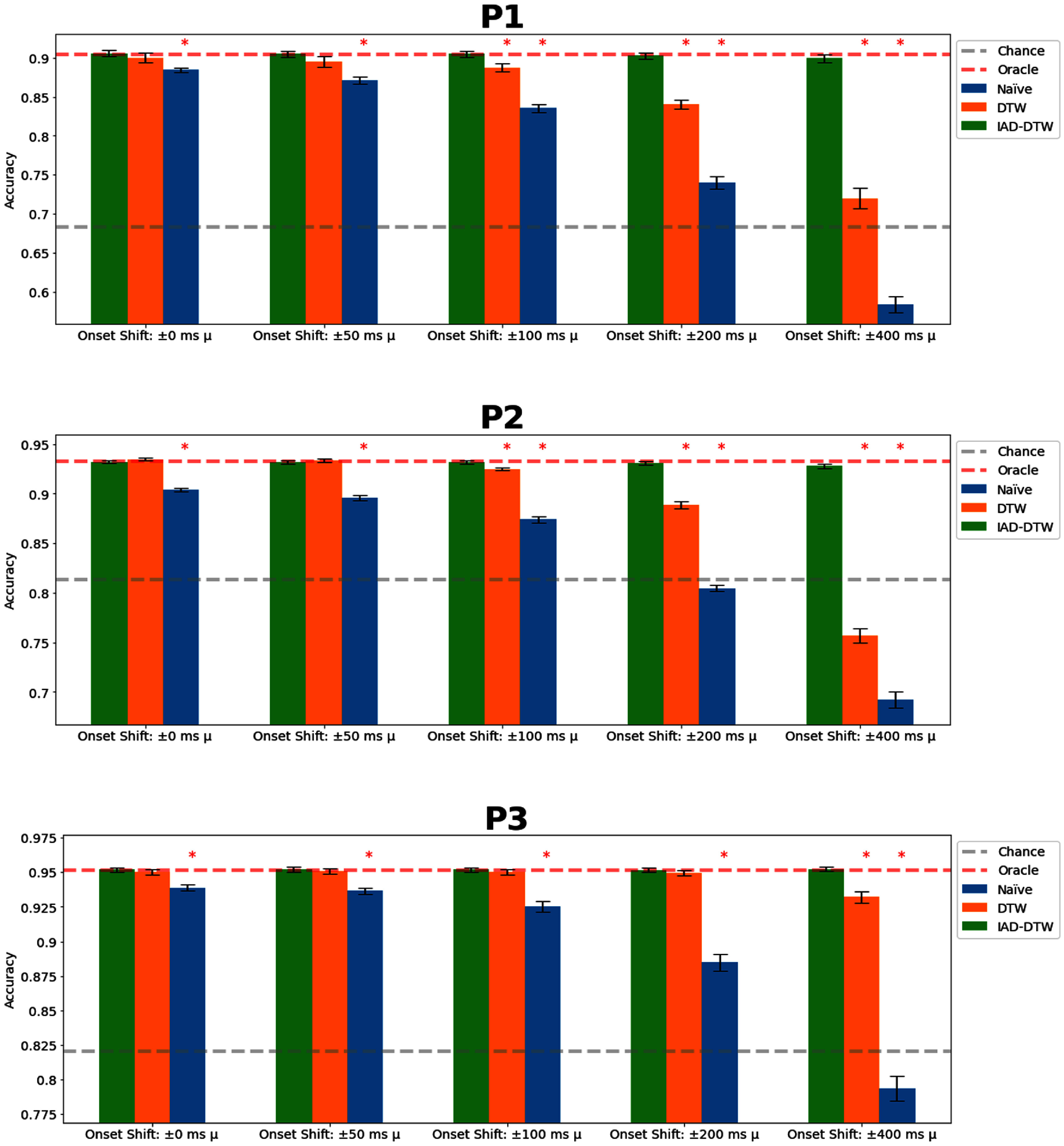
Simulation B—Global label shift. Here, we measure the robustness of naïve (standard, direct classification), dynamic time warping (DTW) and iterative alignment DTW (IAD-DTW) to increasing amounts of unknown shift with respect to the response distribution with a constant, moderate standard deviation of 100 ms and 50 ms in the onset and duration. Statistically significant differences in performance relative to IAD-DTW (ANOVA, *p*-value ⩽ 0.01) are marked by asterisks.

**Figure 6.**
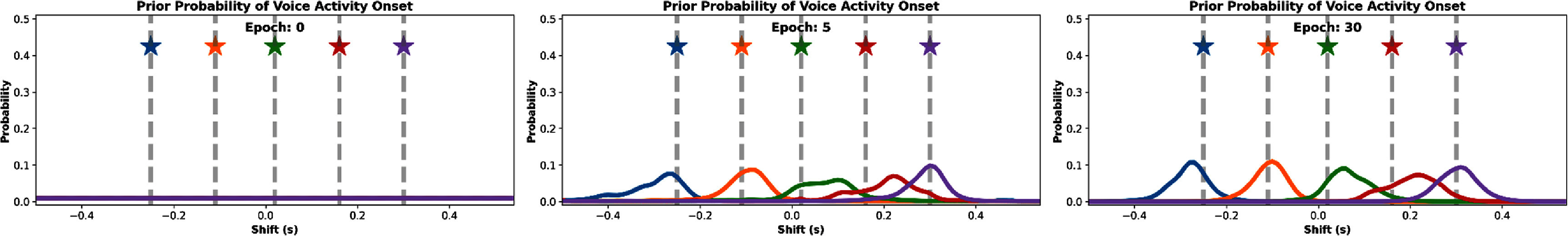
Prior probability convergence. Each colored distribution represents the prior probabilities, or statistical ‘likelihood,’ of a specific shift in onset for aligning neural voice activity for a given trial (randomly selected) at a given epoch during DNN model training. The dotted lines and identically colored stars indicate the unseen ground-truth onsets of voice activity, which are used solely for evaluation. By observing the movement of the distributions and their peaks towards the marked ground truths, we can track convergence of these priors during model training. An animated illustration of this is available in the supplementary materials.

In our second simulation (figure 5), we simulated global shifts in the labels, keeping the jitter fixed to moderate 100 ms onset jitter and 50 ms duration jitter. Unlike random jitter, a consistent unknown bias in response latency impacted even DTW, which resulted in an increased distance from diagonal alignment. The degradation even went so far as to dip (by as much as ∼15%) below even chance levels (achievable via an all-silence classifier). This was because the network consistently attempted to predict mismatching, temporally shifted data. These predictions were visibly noisier and significantly less reliable, as expected from a classifier trying to predict short and inconsistent segments of speech during silence. This can be seen more clearly in figure 6.

### 3.2. Visualization of model learning

In figure 6, we see key snapshots of the average prior probabilities across repeated runs for a single fold. As the model training epochs progressed, we observed that the prior probabilities of our proposed iterative realignment algorithm quickly converged to the true onsets of activity, despite starting from a uniform distribution, as can be seen by the closeness of the peak in each trial’s distribution to the true onset. As network training was stochastic, the learned voice activity onsets necessarily fluctuated to some degree. Nonetheless, these priors tended to plausibly center around the unseen ground-truth onsets. These fluctuations were likely due to a combination of stochasticity during network training and mismatch in label duration (as duration was not yet adjusted) leading to the possibility of multiple optimal shifts. The initial priors could even be entirely wrong, but with our constraints, they consistently moved toward reasonable positions.

**Figure 7.**
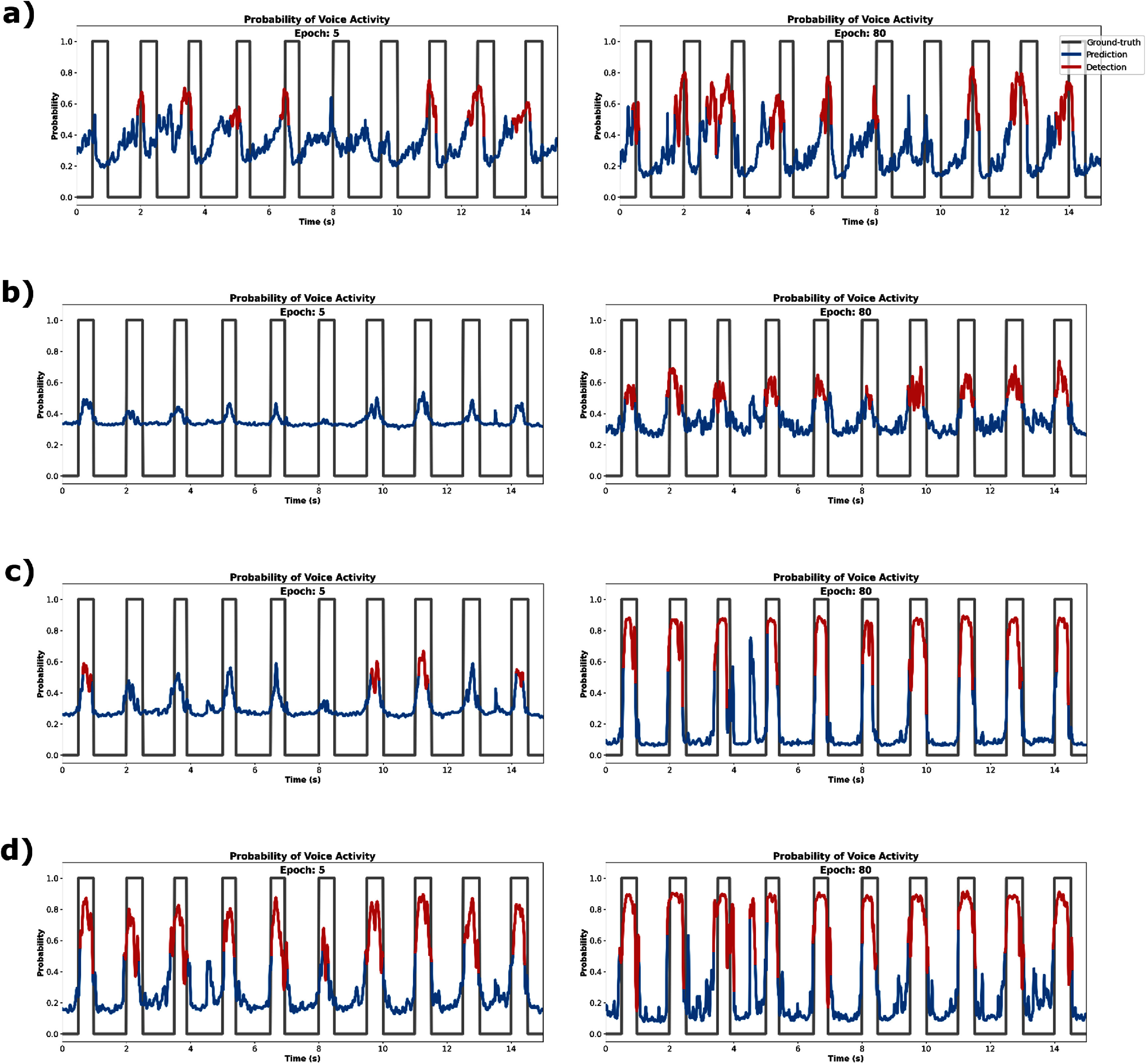
Voice activity probability convergence. The influence of various simulation types on the convergence of learned voice activity with a fixed deep neural network (DNN) architecture (using identical hyperparameters) is illustrated in each panel (a)–(d). Each panel displays ground-truth label traces (gray) and network predictions (blue), along with detections (red) based on filtering the network predictions, in the early (left) and late (right) stages of the training process. In both (a) and (b), the DNN is trained naïvely on labels with unknown shifts, lacking access to a realignment mechanism. Differences in convergence are determined by whether the shifts are (a) global (gross temporal shifts common across all trials) or (b) local (smaller random shifts in the timing of speech labels). In (c), we observe the impact of using our proposed iterative alignment discovery dynamic time warping (IAD-DTW) algorithm under the strain of both global and local shifts. Lastly (d), we show the convergence under the oracle condition, in which the labels are known, and the model is trained naïvely without the need for realignment. Confidence in the predictions is reflected by how square-wave-like the predictions become later within model training. Note that after 80 epochs, IAD-DTW (c) converges on speech probabilities very similar to those under the oracle condition (d). Animated versions of the panels within the illustration are available in the supplementary materials.

In figure 7, we see key snapshots of the evolution of the network predictions over time with respect to the ground truth using IAD-DTW. During training, appropriately trained networks rapidly converge on the final probabilities within the first 5–10 epochs, showing distinct periods of voice activity. On the other hand, jitter without shift resulted in a less confident network, as can be seen by the noisier probability traces even late in model training. Though the probability mass was still centered on voice activity, the thresholded result was less reliable for determining accurate label boundaries. Conversely, a global shift in the labels resulted in not only noisier but also more unreliable probabilities. All these issues were effectively addressed by our IAD-DTW procedure. Even under the most severe label perturbations tested, the performances converged to the level if the labels had been known. Predictions exhibited significantly less noise and higher confidence, resembling a more square-wave-like pattern, akin to the oracle condition. Upon inspecting the filtered detections, we observed that the learned output could effectively segment the voicing duration, as visible in the figure. When labels are mismatched without a correction mechanism, the usability of detections decreases, in alignment with the decrease in confidence of the raw predictions.

### 3.3. Evaluation on unperturbed data

To validate the general applicability of IAD-DTW to data collected in the usual manner—specifically, trials of cued data with the patient’s original, real-world response statistics within the task—we used P3’s untouched neural data, which was available to us. This dataset was divided into 120 trials of visually cued speech, known as ‘syllable reading,’ and 120 trials of auditorily cued speech, known as ‘syllable repetition.’ While we would not anticipate drastic differences in the neural data underlying the motor execution of speech, the timing statistics varied due to differing reaction times to each cue. To address this, we conducted a two-way analysis, training on one half of the data and evaluating on the other half, consistent with the original study from which the data was derived [5]. The only adjustment was the clipping of trials to the same lengths as in P3’s prior analyses, ensuring comparable chance and oracle levels. As in previous analyses, we ran our IAD-DTW procedure five times for each of 5 cross-validation folds, resulting in 25 values, to generate confidence bounds for the resulting accuracies. Here, we assessed performance on the alternate dataset instead of the held-out fold to provide a more rigorous test of model generalization. We observed accuracies for reading and repetition (95.1 ± 0.3% and 95.2 ± 0.2%, respectively) that were comparable to those in the oracle condition for P3 (95.0% and 95.1% accuracy, respectively), where labels were known.

## 4. Discussion

The purpose of our investigation is not to locate speech freely within unconstrained periods, but rather to refine the precise temporal boundaries of data collected for model training under controlled conditions. The simplest and most straightforward approach in this context is to train a DNN (or any supervised model) using ordinary backpropagation to predict hand-selected speech boundaries for each trial of neural data. This represents a ‘naïve model,’ which relies on the assumption that the manually labeled training targets (here the onsets and durations of speech) are entirely correct and accurate. However, in practice, doing so may be infeasible or even impossible in certain patient populations.

This problem motivates our approach, which is designed to discover the timing of neural activity associated with speech production in patients attempting to speak in response to a computer prompt, while accounting for variations in response latencies and durations. This approach differs most significantly from previous studies, where patients spoke within experimentally enforced response periods or synchronized their responses with predictable cues [21] that implicitly provide an alignment or segmentation with relevant neural activity. While previous studies used experimental timing constraints alone to facilitate the training and convergence of a recurrent neural network, our approach jointly and flexibly discovers the onsets and durations of neural voice activity starting only with an estimate of the median onset latency and duration of speech produced in response to instructional cues.

Detecting high gamma activity based solely on energy levels, such as through peak detection, is a challenging and inconsistent task. Typically, training-free methods like these depend on techniques such as smoothing, channel weighting and basic task/active control context to achieve reliable control context to achieve reliable nVAD, even in the case of overt speech [5, 42]. One promising alternative strategy for covert control without the need for external behavioral timing cues has also been investigated, in which explicit neural activity templates are optimized based on known statistical priors on the onset, duration and temporal profile of attempts [14]. On the other hand, our method progressively adjusts and improves the low-dimensional prediction target directly. This is particularly useful in patients with LIS, for whom obtaining such templates can be challenging. Additionally, our approach simplifies model optimization by focusing on labeling neural voice activity rather than modeling the typically noisy, highly correlated, and high-dimensional input neural features directly. While recent work has shown promising results in speech decoding using MEAs [14, 19, 22], the long-term stability of MEAs for speech decoding must be characterized. Regardless, the higher signal-to-noise ratio (SNR) of spiking neural signals during peak device functionality may not require as precise of time-alignment cues for model convergence.

As speech BCIs evolve to deliver more naturalistic and immediate output for improved communication, the methods for training the neural decoding models powering these systems must adapt accordingly. The impact of mismatches between speech BCI output and silent speech attempts remains uncertain. However, the necessity for a temporally precise ground truth signal is arguably most pronounced in real-time speech synthesis applications, where low latencies and tight synchronization are critical both for model training and for real-time model performance. Delayed [43] and perturbed [44] auditory feedback has been shown to negatively impact speech fluency in able-bodied people and has been regulated via audio-visual synchronization standards in visual media [45] and real-time teleconferencing [46]. While immediate, visual-only, textual output at the level of letters and words may tolerate higher levels of misalignment, accumulating evidence across studies of acoustic feedback emphasizes the benefits of its synchronization with speech production at timescales matching that of phonemes [25–27].

In this study, we evaluated the effectiveness of our approach compared to alternative methods when voice activity occurred at any point or duration within a trial of ECoG, with weak statistical assumptions about typical speech responses that assume that the data was collected in a typical cue-response paradigm for BCI model training. Prior to our investigations, the impact of uncertainty about the timing of attempted speech on nVAD model performance had not yet been characterized. Such scenarios are typical in training speech BCI decoders for patients with LIS, as it is difficult to ascertain their ability to reliably modulate neural activity within constrained time periods. Patients with LIS may have difficulty attempting certain articulations, and their response latencies may vary due to ebbs and flows in energy and attention and variations in task difficulty. Lack of auditory feedback adds to the difficulty of consistently constraining the timing of attempted speech and its corresponding neural activity.

We systematically tested such degradations by manipulating the location and level of temporal jitter of utterances. Our simulations revealed that traditional methods relying on blind labeling performed moderately well, often far above chance accuracy, when the temporal overlap between corrupted and true labels was high (⩾50%). However, when this overlap was low (<50%), model performance with traditional methods plummeted to chance or even sub-chance levels. Particularly, global shifts of around 200 ms led to such degradation, where corrupted labels were nearly or entirely non-overlapping temporally with their true positions.

In contrast, when using our IAD-DTW approach, we achieved nearly oracle level performance regardless of the degree of label corruption. This improved performance was primarily due to our Slide-DTW method’s label relocation mechanism, which was further reinforced by our stochastic EMA tying method. This ensured that only the most consistent labels for each trial across progressive training epochs won out as the network progressively converged to a final set of timing labels. In contrast, traditional methods relied only on a possibly severely shifted frame of reference, requiring the prediction of speech from periods of silence by overfitting to background neural activity. The absence of a flexible but informed relocation mechanism can lead to an ‘avalanche effect,’ in which the model is progressively trapped in a local minimum. The EMA tying mechanism helped steer the model towards a consistent alignment hypothesis while discouraging it from latching onto spurious alignments due to random noise within the data or due to bad initializations.

Notably, even standard DTW struggled to correct gross temporal shifts, because off-diagonal movements (or warps) were implicitly penalized. DTW paths were summed, and unless a strong prior already existed, DTW alone was unlikely to deviate from its bias toward a simple one-to-one diagonal alignment. Given the randomness of network initialization and the high levels of noise in the data, such a strong statistical prior was unlikely to emerge without further guidance.

A key limitation of our study is that our simulations were derived from patients who could still speak and who did so during the experiment. This choice was made to have a reliable reference for benchmarking our method against standard approaches. Even in complete paralysis, motor networks remain intact, reflected in motor activations during attempted movements closely resembling those during actual movements [47, 48]. In contrast, imagined movements result in far less cortical activation, even in individuals who are no longer able to actually move [47, 48]. This motivates the study of analogous motor networks for speech. While speaking aloud can activate cortex in both auditory [49, 50] and motor [49–51] areas during self-perception of one’s own speech, which may in turn contribute to decoding, studies have demonstrated that the level of remaining activation during silent speech articulation is sufficient for a high-performing speech BCI [17], a prerequisite for IAD-DTW. Further research may be needed to further establish the feasibility of truly silent speech decoding in clinical trial patients. Participants in studies to date could still phonate to some degree, albeit unintelligibly [21–23]. The minimal and nonspecific auditory feedback in these clinical trial patients suggests the feasibility of training silent speech decoders without normal auditory feedback.

Another notable limitation is that our approach is not tailored for naturalistic or self-paced speech lacking externally cued pauses. While we acknowledge this constraint, our procedure aligns with the common method for collecting training data for a BCI—within the structured framework of trials. As IAD-DTW pertains only to model training, the usefulness of the trained neural decoder is limited by the diversity of the data used to train it and how readily the model is structured for real-time operation.

The transferability of IAD-DTW to attempted speech is expected to depend on the SNR and consistency of the neural activity produced by speech attempts. If speech attempts vary widely in latency and duration compared to the actual speech productions used in this study, it may be challenging for the training model to distinguish between actual speech responses and noise. Additionally, if the SNR of the task-modulated neural activity is too low, IAD-DTW will struggle to converge even if the labels are known. Thus, the task paradigms for which IAD-DTW is designed need to evoke neural responses with sufficient strength to be distinguishable from background fluctuations and consistent enough for natural behavioral response statistics to be leveraged. Future work may improve our approach to address these limitations and expand IAD-DTW’s applicability in more diverse clinical settings, specifically as it pertains to attempted speech.

## 5. Conclusion

IAD-DTW represents a promising step forward in identifying the timing of speech-associated neural activity when spoken acoustics are not available. Our method demonstrates that even limited access to statistical priors on the timing of attempted speech, such as the number of utterances and expected response latencies and durations, can provide useful information for detecting speech-associated neural activity. While our approach has limitations for cases in which response latencies are highly variable and SNRs are low, even approximate labels can be valuable in situations in which audible outputs are unavailable and speech attempts cannot be directly observed. Furthermore, IAD-DTW has the potential to serve as a useful automated initialization for supervised model training, even when acoustic outputs are available. Future work will explore how to extend our approach to accommodate more complex speech tasks and to overcome its current limitations to further advance the field of speech decoding in individuals with impaired or absent speech due to impaired motor output.

## Data Availability

No new data were created or analyzed in this study.

## Data access

The data used in this study is publicly available from https://figshare.com/collections/Human_ECoG_speaking_consonant-vowel_syllables/4617263 and https://gigantum.com/griffinmilsap/ecog-keyword-spotting.

## Code availability

The code to reproduce the core methods used in this study is publicly available from https://github.com/cronelab/IAD-DTW.
